# Acute Pancreatitis as a Possible Unusual Manifestation of COVID-19 in Children

**DOI:** 10.1155/2021/6616211

**Published:** 2021-02-02

**Authors:** Niloufar Bineshfar, Alireza Mirahmadi, Fereshteh Karbasian, Elham Pourbakhtyaran, Abdollah Karimi, Mehdi Sarafi

**Affiliations:** ^1^Student Research Committee, School of Medicine, Shahid Beheshti University of Medical Sciences, Tehran, Iran; ^2^Department of Pediatrics Emergency, Mofid Children's Hospital, Shahid Beheshti University of Medical Sciences, Tehran, Iran; ^3^Pediatric Specialist, Emergency Department, Shahid Beheshti University of Medical Sciences, Tehran, Iran; ^4^Pediatric Infections Research Center, Mofid Children's Hospital, Shahid Beheshti University of Medical Sciences, Tehran, Iran; ^5^Pediatric Surgery Research Center, Research Institute for Children's Health, Shahid Beheshti University of Medical Sciences, Tehran, Iran

## Abstract

Coronavirus disease-2019 (COVID-19) which is caused by severe acute respiratory syndrome coronavirus-2 (SARS-CoV-2) has spread throughout the world causing problems for millions of people. Symptoms of COVID-19 in pediatric patients include both respiratory and gastrointestinal symptoms. The most common symptoms are fever, cough, and fatigue. In this report, we describe a case of a previously well 14-year-old boy, who presented to our emergency department with a complaint of abdominal pain, nausea, and vomiting without fever or respiratory symptoms. He was diagnosed with acute pancreatitis based on an abnormal amylase level and abdomen computed tomography (CT) and later found to be infected by SARS-CoV-2, by a positive reverse transcriptase-polymerase chain reaction (RT-PCR) test.

## 1. Introduction

In December 2019, severe acute respiratory syndrome coronavirus-2 (SARS-CoV-2), which causes coronavirus disease-2019 (COVID-19), was discovered in Wuhan, China [1].

COVID-19 in children presented mostly with mild upper respiratory symptoms. The most common symptoms are fever, cough, and fatigue; however, gastrointestinal symptoms such as diarrhea, vomiting, and abdominal pain were also reported [2, 3].

Acute pancreatitis (AP) which is caused by medications, trauma, gallstones, and less prevalently viral infections in pediatric patients is the sudden inflammation of the pancreas [4, 5]. We report a case of a COVID-19-associated pancreatitis presenting in a 14-year-old boy.

## 2. Case Report

A 14-year-old boy presented to our emergency department with abdominal pain in the epigastric region associated with anorexia, nausea, and vomiting for 24 hours. The patient had no fever or respiratory symptoms. There was no underlying disease. He mentioned a history of contact with his aunt who was suspected of COVID-19. On physical examination, the patient was not ill or toxic and vital signs were normal: RR: 20, PR: 98, BP: 100/65, and T: 36.9°C. The abdomen was soft, nondistended with right lower quadrant and epigastric tenderness.

**Figure 1:**
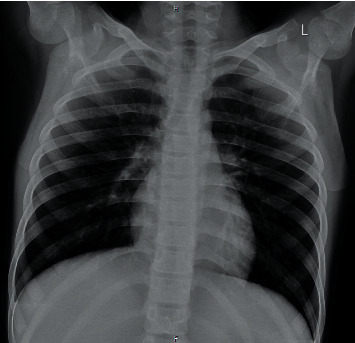
Chest X-ray.

**Figure 2:**
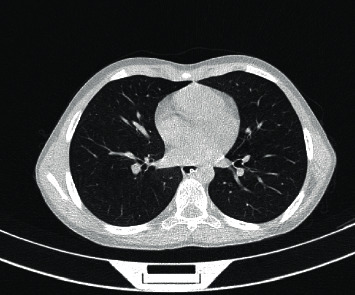
Chest CT.

**Figure 3:**
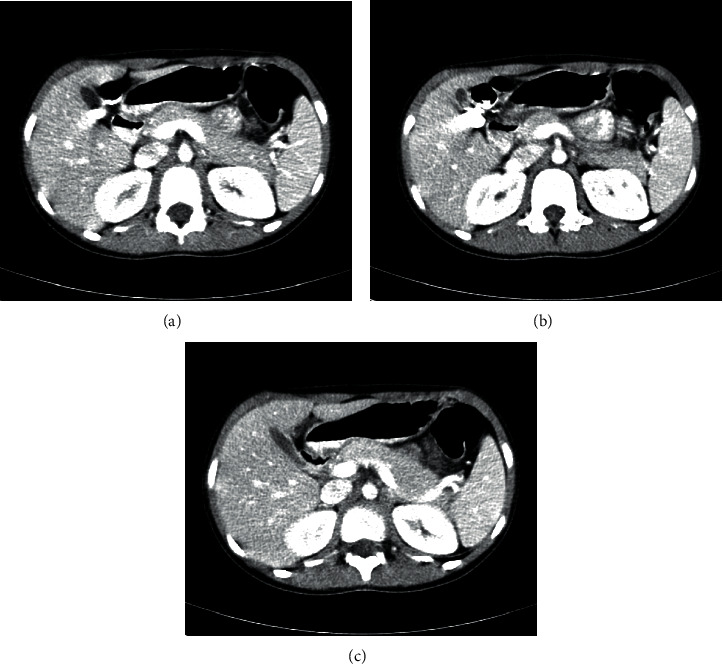
Abdomen CT.

Laboratory tests revealed elevated amylase (1914, normal <100 *μ*/l) and lymphopenia, and other blood test results were within normal limits (Table 1). Later, a nasopharyngeal swab specimen was collected which tested positive for SARS-CoV-2 on reverse transcriptase-polymerase chain reaction (RT-PCR). Due to the positive SARS-CoV-2 RT-PCR, a chest X-ray (Figure 1) and computed tomography (CT) (Figure 2) were obtained. Abdomen CT was suggestive of pancreatitis (Figure 3).

The patient was treated with bowel rest, intravenous crystalloid fluid resuscitation, and ondansetron, pantoprazole, and empiric antibiotics including ceftriaxone and metronidazole. The symptoms were resolved gradually within 3 days, and the patient was discharged with decreasing amylase level.

## 3. Discussion

AP in pediatric patients is most commonly caused by drugs, trauma, and gallstones [4, 5]. Less common causes of AP have been reported as infections. The relation between AP and some viruses such as CMV, HIV, HSV, EBV, VZV, mumps virus, coxsackievirus, and some others has been proven [4–7]. Based on the Revised Atlanta Classification System for AP Diagnosis, at least two of the three following criteria should be found: (1) abdominal pain (defined as acute onset, persistent, severe epigastric pain often radiating to the patients' back), (2) increased serum lipase or amylase levels to greater than 3 times the upper limit of normal value, and (3) characteristic findings of AP on contrast-enhanced CT [8]. AP was diagnosed in our patient based on the Atlanta criteria. In the search for etiology, none of the common causes of AP was found and the only significant finding was a positive SARS-CoV-2 PCR test. Recent studies have shown gastrointestinal symptoms are fairly common in COVID-19 but the relation between COVID-19 and AP has not been described [9–11]. However, a case series of 52 COVID-19 patients reported pancreas injury in 9 patients (17% of patients) [12]. The clear pathogenesis of AP in COVID-19 patients is unknown, but the mechanism of AP following viral infections is different and depends on the type of the virus involved [5]. Currently, there is some evidence showing SARS-CoV-2 enters cells in the lungs and gastrointestinal system by binding to the angiotensin-converting enzyme 2 (ACE2) (and transmembrane protease, serine 2 (TMPRSS2) receptors), as another study by using immunostaining has shown ACE2 is highly expressed in the pancreas; thus, AP in COVID-19 patients could occur due to the direct cytopathic effect of local virus replication [12, 13]. The endothelial location of the ACE2 receptors and response to SARS-CoV-2 infection may cause increased thrombophilia in pancreas vessels in COVID-19 patients, and this vascular thrombosis may lead to AP [14]. Also, AP may be caused by the indirect effect of the harmful systemic immune response induced by SARS-CoV-2 infection [12]. A certain association between COVID-19 and AP has not been proved yet so we recommend further research to be conducted to evaluate any possible relation. A study reported serum lipase level rising in COVID-19 patients without AP symptoms [15]. Therefore, we suggest that AP diagnosis should be based on Atlanta criteria and not only on the serum lipase level. In addition, the serum lipase level may not be specific and can rise in other diseases and conditions including increased gut permeability (diarrhea) with SARS-CoV-2 infection [16, 17]. Considering the possible association between COVID-19 and AP, we suggest full personal protective equipment (PPE) in contact with patients with AP symptoms and also in COVID-19 recognition test if possible.

## Figures and Tables

**Table 1:** Laboratory results.

Laboratory results	Day 1	Day 2	Day 3
White cell count	23.9	9.3	10.3

Differential count			

Neutrophils	22466	7877	8034
Lymphocytes	956	912	1545
Hemoglobin	13.9	9.6	11.7
Hematocrit	41.4	30.5	36.4
Platelet count	369	184	217
ALT	38	20	
AST	33	15	
Alkaline phosphatase	328	232	
Amylase	1914	200	
Blood sugar	120		94
Blood urea nitrogen	14.3		5.6
Creatinine	0.8		0.56
C-reactive protein	4	4	11
Na	140		138
K	4.5		4.5
Erythrocyte sedimentation rate	18	26	42

## Abbreviations

SARS-CoV-2: Severe acute respiratory syndrome coronavirus-2
COVID-19: Coronavirus disease-2019.
